# Characterisation of meropenem‐resistant *Bacillus* sp. FW 1 isolated from biogas digestate

**DOI:** 10.1111/1758-2229.13217

**Published:** 2023-11-15

**Authors:** He Sun, Jolanta J. Levenfors, Christian Brandt, Anna Schnürer

**Affiliations:** ^1^ Department of Molecular Sciences Swedish University of Agricultural Sciences Uppsala Sweden; ^2^ Ultupharma AB Uppsala Sweden; ^3^ Institute for Infectious Diseases and Infection Control Jena University Hospital Jena Germany

## Abstract

Recently a *Bacillus* sp. strain FW 1 was isolated from biogas digestate and shown to have novel resistance to meropenem (MEM), of critical importance in human medicine. MEM‐resistance has so far only been described for one species within the genus *Bacillus*, that is, *Bacillus cereus*. *Bacillus* is an abundant representative of the microbial community in biogas digesters and consequently, the finding indicates a risk of spreading such resistance when using the digestate as fertiliser. In this study, the *Bacillus* strain was characterised and classified as *Heyndrickxia oleronia* (*previous Bacillus oleronius*), previously not described to harbour MEM‐resistance. The mechanism of resistance was explored by metallo‐β‐lactamase (MBL) production, mapping of carbapenemase genes and genome analysis. The transferability of MEM‐resistance in strain FW 1 was investigated by plasmid transformation/conjugation, combined with genome analysis. The results confirmed MBL production for both strain FW 1 and the type strain *H. oleronia* DSM 9356^T^. However, elevated MEM resistance was found for strain FW 1, which was suggested to be caused by the production of unclassified carbapenemase, or overexpression of MBL. Moreover, the results suggest that the MEM‐resistance of strain FW 1 is not transferable, thus representing a limited risk of MEM‐resistance spread to the environment when using digestate on arable land.

## Graphical abstract

The novel resistance of *Heyndrickxia oleronia* (formerly known as *Bacillus oleronius*) FW 1 to meropenem is suggested to arise from the production of unclassified carbapenemase, or an overexpression of metallo‐β‐lactamase. It is suggested that the meropenem resistance observed in strain FW 1 is not transferable.

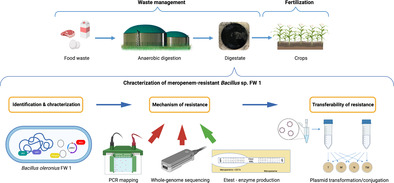

## INTRODUCTION

Antibiotic resistance is one of the most significant global public health challenges of our time (CDC, 2019). Annually, infections with antibiotic‐resistant pathogens account for over 30,000 attributable deaths in Europe (Cassini et al., 2019) and the United States (CDC, 2019). Important steps in addressing the antibiotic resistance threat are to prevent infections in the first place and to reduce the transfer of antibiotic resistance to pathogens. Anaerobic digestion (AD) represents a potential source of the spread of antibiotic resistance (Youngquist et al., 2016). During AD, various municipal and industrial organic waste streams or energy crops/crop residues are used to produce biogas and digestates (Schnürer & Jarvis, 2018). Biogas can be used for the production of electricity, heat, or vehicle fuel, and the digestate, which is rich in plant nutrients, is commonly used as a biofertiliser on arable land, creating a potential route for the spread of antibiotic resistance (Corden et al., 2019; Kougias & Angelidaki, 2018). However, the use of digestate in agriculture is highly beneficial, as it contributes to the recycling of mineral nutrients (N, P, K, etc.) between urban and rural areas, adds organic carbon to the soil and replaces fossil‐demanding conventional chemical fertilisers (Risberg, 2015). AD is a key process for the development of a fossil‐independent society and thus an increase in the number of biogas plants is predicted, so the use of digestate as a biofertiliser is expected to increase significantly in the short to medium term (Corden et al., 2019).

Substrates entering the biogas system and the resulting digestate have been found to contain antibiotic‐resistance components, including antibiotic residues, antibiotic‐resistant bacteria (ARB) and antibiotic‐resistance genes (ARGs) related to a variety of antibiotic agents (Luo et al., 2017; Schauss et al., 2016; Sun et al., 2022; Youngquist et al., 2016). Among different antibiotics, carbapenems and cephalosporins (third and fourth generation), belonging to β‐lactam antibiotics, are of particular concern as regards resistance, because they are critically important for human medicine according to a ranking of antibiotics by the World Health Organization (Collignon et al., 2016). Carbapenems are also considered the most reliable last‐resort treatment for multidrug‐resistant bacterial infections (Meletis, 2016). Resistance to carbapenem is unfortunately spreading globally, mainly via pathogens in clinical sectors (Meletis et al., 2015; Walsh et al., 2005). However, recent studies have identified carbapenemase genes in different waste streams, such as *bla*_VIM_ in digestate originating from cattle manure (Ma et al., 2019) and *bla*_KPC‐2_ in sludge from a wastewater treatment plant (WWTP) (Yang et al., 2016). Their corresponding enzymes VIM and KPC are highly effective carbapenemases, in terms of both carbapenem hydrolysis and geographical spread (Meletis, 2016). Moreover, recently a bacterial strain (further investigated in this paper) belonging to the genus *Bacillus*, isolated from digestate derived from food waste, was found to display resistance to meropenem (MEM), an agent of carbapenems (Sun, Bjerketorp, et al., 2020). *Bacillus* species are often intrinsically resistant to β‐lactams as a consequence of the intrinsic production of penicillinase, cephalosporinases and carbapenemases (Chen et al., 2003; Torkar & Bedenić, 2018). However, members within this genus are mostly susceptible to carbapenems, even though metallo‐β‐lactamases (MBLs) genes such as *Bla*2 in *Bacillus anthracis* (Chen et al., 2003) and *Bc*II in *Bacillus cereus* (Baldwin et al., 1980) are carried on some of their chromosomes. To our knowledge, carbapenem resistance has before only been described for one species within the genus *Bacillus*, that is *B. cereus*, but without investigation of resistance mechanisms (Savini et al., 2009).

Antibiotic resistance, especially carbapenem resistance associated with *Bacillus* is worrying since this genus is commonly detected in biogas processes. *Bacillus* is suggested to play a role in the conversion of fat and carbohydrates to acids during the AD process (Tao et al., 2020). It is an abundant genus in biogas digestate, as indicated by both bacterial community analysis and culture‐dependent isolation (Schauss et al., 2016; Tao et al., 2020). The genus is a member of the core bacterial microbiota prevailing in various types of biogas reactors, with a relative abundance of over 10% according to pyrosequencing (Tao et al., 2020). In a study analysing output samples from 15 biogas plants operating with animal manures and plant silage, in mesophilic or thermophilic conditions, it was found that 70.9% of a total of 902 isolates could be assigned to *Bacillus* (Schauss et al., 2016). In another study, *Bacillus* and closely related genera, for example, *Paenibacillus*, *Lysinibacillus* and *Brevibacillus*, were found to be the dominant members of an ARB community isolated from the digestate of reactors processing animal manure and food waste (Sun, Bjerketorp, et al., 2020). The bacteria isolated belonging to these genera exhibited resistance to a variety of antibiotic classes, including tetracycline (TET), β‐lactams, macrolides and so forth, and therefore appeared to represent a group of bacteria within the biogas process exhibiting broad antibiotic resistance (Sun, Bjerketorp, et al., 2020). In addition, *Paenibacillus* spp. isolated from a WWTP have been found to carry a carbapenemase gene *bla*_KPC‐2_ (Yang et al., 2016). Members in *Bacillus* are also active in horizontal gene transfer (HGT) through plasmids transformation to other *Bacillus* species (Bernhard et al., 1978) and even with other genera such as *Staphylococcus aureus* (Canosi et al., 1978) and *Escherichia coli* (Zhao et al., 2020). Therefore, if the carbapenemase gene is located on mobile genetic elements, for example plasmids, transposons and integrons, there is a risk of spreading to other non‐resistant *Bacillus* spp. and other bacterial genera. In this regard, the previously isolated MEM‐resistant *Bacillus* strain (Sun, Bjerketorp, et al., 2020) could indicate a risk of carbapenem resistance spreading within biogas plants, and to the environment when the digestate is applied as biofertiliser.

Few carbapenem‐resistant bacterial strains have been isolated from digestates so far (Schauss et al., 2016), and the *Bacillus* sp. isolated from the digestate is the first known carbapenem‐resistant *Bacillus* isolate besides *B. cereus*. Thus the isolated *Bacillus* strain is of particular importance in revealing information on hosts of carbapenem resistance and corresponding resistance mechanisms, and most importantly the possibility of resistance transfer in digestate. Resistance transferability would indicate risk to the environment when using digestates as fertiliser. Certain *Bacillus* spp. are highly similar in terms of 16S rRNA gene sequence. Thus it is often impossible to identify them precisely with only 16S rRNA gene similarity, and polyphasic approaches, for example biochemical tests, specific DNA probes and whole‐genome sequence (WGS) comparison are often co‐applied for accurate identification (Blackwood et al., 2004; Giffel et al., 1997). The aims of this study were, therefore, to characterise and taxonomically position the MEM‐resistant *Bacillus* strain FW 1, through comparisons of WGS and phenotypic characteristics; to obtain information on its mechanism of resistance by primer‐specific PCR mapping, WGS analysis and carbapenemase production tests; and to investigate the transferability of MEM resistance through genome analysis and plasmid transformation/conjugation tests.

## EXPERIMENTAL PROCEDURES

### Samples

The MEM‐resistant strain of *Bacillus* was originally isolated from a laboratory‐scale mesophilic biogas reactor operating with food waste (Sun, Bjerketorp, et al., 2020). The food waste was derived from a commercial‐scale biogas plant in Uppsala, Sweden. Information regarding the operation and inoculum of the reactor can be found in a previous work (Westerholm et al., 2015). In this paper, the strain is referred to as FW 1 (isolated strain No. 1). Details of isolation and antibiotic resistance pattern for strain FW 1 can be found in Sun, Bjerketorp, et al. (2020). In this work, FW 1 was revived from glycerol (25% v/v) stock frozen at −80°C and cultivated in Mueller Hinton broth (MHB, Becton Dickinson) at 37°C. In addition, the type strain *Heyndrickxia oleronia* DSM 9356^T^ was obtained from the German Collection of Microorganisms and Cell Cultures GmbH (DSMZ).

### Long‐read DNA sequencing

#### DNA preparation

For both FW 1 and *H. oleronia* DSM 9356^T^, long fragmented genomic DNA was extracted using NucleoBond kit (Macharey Nagal) and purified using AMPure magnetic beads (Beckman Coulter) according to the protocol described in Sun, Brandt, and Schnürer (2020). The DNA concentration was quantified by Qubit (Thermo Fisher Scientific), and the fragment length was visualised by agarose gel electrophoresis.

#### Sequencing

SQK‐LKS109 and EXP‐NBD104 kits (Oxford Nanopore Technologies) were used for library preparation, according to the manufacturer's instructions. Long‐read sequencing was performed using a MinION device (Oxford Nanopore Technologies) for 72 h at a bias voltage of −180 mV, with a FLO‐MIN106 flow cell. Refuelling using a ‘Refuel‐Mix’ was performed after the first 18 h of sequencing.

#### Sequence processing

Raw nanopore sequencing data were basecalled and demultiplexed using guppy (v. 4.0.15–1) and filtered by filtlong (v. 0.2.0). Genome reconstruction was performed using flye (v. 2.8) and subsequently polished using racon (v. 1.4.13) and medaka (v. 1.0.3). Read mapping for polishing was performed using minmap2 (v. 2.17). Assemblies were submitted to NCBI, with accession number CP079720 for strain FW 1 chromosome CP079721–CP079723 for its extra‐chromosomal DNA (ecDNAs), and CP080028 for *H. oleronia* DSM 9356^T^ chromosome.

### Strain FW 1 identification and characterisation

#### Phenotypic characteristic tests

Biochemical tests were carried out on API 20E and 50CHB arrays (BioMérieux, Sweden AB) according to the manufacturer's instructions. The pH range for growth at 37°C was determined from 4.0 to 12.0 in steps of 1.0 pH unit in MHB, adjusted with HCl (Sigma‐Aldrich) and NaOH (VWR Chemicals BDH) solutions (1 M for each) after filtration sterilisation (SARSTEDT AG & Co. KG). The temperature range for growth was tested at 20, 30, 37 and 55°C in MHB. In addition, anaerobic growth was tested using anaerobic jars with deoxygenates (Merck KGaA). Bacterial growth was quantified by a WPA CO8000 cell density meter (Biochrom) after overnight incubation. Antibiotic resistance pattern profiling was performed following the protocol described by Sun, Bjerketorp, et al. (2020).

#### Determination of genome sequence similarity

A comparison of WGS similarity was performed for strain FW 1, *H. oleronia* DSM 9356^T^, and *Heyndrickxia Sporothermodurans* DSM 10599^T^ using the sequences of strain FW 1 and *H. oleronia* DSM 9356^T^obtained from Oxford Nanopore sequencing (Section Long‐read DNA sequencing). For *H. sporothermodurans*, the genome sequence of the type strain DSM 10599^T^ (accession no. GCF_016756695.1) was retrieved from NCBI (cited 1 March 2023). Three algorithms were used to analyse the genome similarity: (a) average nucleotide identity (ANI), measured by OrthoANI (Lee et al., 2016); (b) DNA–DNA hybridisation (DDH), calculated by in silico DDH through the web service at http://ggdc.dsmz.de (Meier‐Kolthoff et al., 2013); and (c) genome similarity, visualised by Proksee (Stothard & Wishart, 2005).

### Mechanism of carbapenem resistance

#### PCR mapping

##### PCR assay design

In total, 15 groups of globally spread carbapenemase (G1–G3) and extended‐spectrum β‐lactamase (ESBL) (G4–G6) genes were targeted in this work, and five multiplex PCR (G1–G5) and one single‐plex PCR (G6) were designed (Table 1). Rapid DNA preparation was performed from one single colony suspended in a total volume of 100 μL of distilled water (95°C for 10 min). Cell debris was removed by centrifugation for 1 min at 13,000 rpm. Retrieved DNA (2 μL) was exposed to each multiplex and single‐plex PCR group in a 25 μL reaction volume, using the Illustra PuReTaq Ready‐To‐Go PCR Beads kit (GE Healthcare) according to the manufacturer's instructions. Amplification was carried out as follows: initial denaturation at 94°C for 10 min, 30 cycles at 94°C for 40 s, 60°C for 40 s and 72°C for 1 min, and a final elongation step at 72°C for 7 min. For the G3 multiplex PCR group, the annealing temperature for optimal production was 55°C (Dallenne et al., 2010). Amplicons were visualised by electrophoresis running at 100 V for 1 h on 2% agarose gel. GeneRuler 100 bp plus was used as a size marker (Thermo Fisher Scientific Inc.). After purification by AMPure XP (Beckman Coulter, Inc. Brea, CA, USA), and quantification by Qubit (Invitrogen, Thermo Fisher Science, Waltham, MA, USA), the PCR products were sent to Macrogen Europe (Amsterdam, Netherlands) for Sanger sequencing.

**TABLE 1:** Group‐specific primers used for mapping carbapenemase and extended‐spectrum β‐lactamase genes.

Group	Gene group	Genes targeted	Primer name	Sequence (5′‐3′)	Amplicon size (bp)	References
G1	OXA‐40	OXA‐40‐like genes (*n* = 12)	OXA40‐F	GTCCCTGCATCAACATTTAAGATGC	563	Brandt et al., 2016
			OXA40‐R	GTAATTTCATTACGAATAGAACCAG		
	OXA‐48	OXA‐48‐like genes (*n* = 10)	OXA48‐F	GCGTGTATTAGCCTTATCGGCTG	204	Brandt et al., 2016
			OXA48‐R	GCGGGTAAAAATGCTTGGTTCGC		
	OXA‐51	OXA‐51‐like genes (*n* = 106)	OXA51‐F	CGAGTATGTACCTGCTTCGACC	485	Brandt et al., 2016
			OXA51‐R	CAACCCATCCAGTTAACCAGCC		
G2	OXA‐23	OXA‐23‐like genes (*n* = 16)	OXA23‐F	CGCGCAAATACAGAATATGTGCC	152	Brandt et al., 2016
			OXA23‐R	CCTAGTGTCATGTCTTTTTCCCAAG		
	OXA‐58	OXA‐58‐like genes (*n* = 5)	OXA58‐F	GCTGTGTTTGTCACTTATGATGG	514	Brandt et al., 2016
			OXA58‐R	CCATTCCCCAGCCACTTTTAGCATA		
	OXA‐235	OXA‐235‐like genes (*n* = 9)	OXA235‐F	CAAGCCATGCAAGCTTCTGC	305	Brandt et al., 2016
			OXA235‐R	TCCATACCCCAACCAGATTTGGC		
G3	VIM	VIM variants including VIM‐1, VIM‐2 and VIM‐7	VIM‐F	GATGGTGTTTGGTCGCATA	390	Ellington et al., 2007
			VIM‐R	CGAATGCGCAGCACCAG		
	IMP	IMP variants except IMP‐9, IMP‐16, IMP‐18, IMP‐22 and IMP‐25	IMP‐F	TTGACACTCCATTTACDG^a^	139	Dallenne et al., 2010
			IMP‐R	GATYGAGAATTAAGCCACYCT^a^		
	KPC	KPC‐1 to KPC‐5	KPC‐F	CATTCAAGGGCTTTCTTGCTGC	538	Dallenne et al., 2010
			KPC‐R	ACGACGGCATAGTCATTTGC		
G4	TEM	TEM variants including TEM‐1 and TEM‐2	TEM‐F	CATTTCCGTGTCGCCCTTATTC	800	Dallenne et al., 2010
			TEM‐R	CGTTCATCCATAGTTGCCTGAC		
	SHV	SHV variants including SHV‐1	SHV‐F	AGCCGCTTGAGCAAATTAAAC	713	Dallenne et al., 2010
			SHV‐R	ATCCCGCAGATAAATCACCAC		
G5	CTX‐M‐group 1	CTX‐M‐1, CTX‐M‐3 and CTX‐M‐15	CTXMG1‐F	TTAGGAARTGTGCCGCTGYA^a^	688	Dallenne et al., 2010
			CTXMG1‐R	CGATATCGTTGGTGGTRCCAT^a^		
	CTX‐M‐group 2	CTX‐M‐2	CTXMG2‐F	CGTTAACGGCACGATGAC	404	Dallenne et al., 2010
			CTXMG2‐R	CGATATCGTTGGTGGTRCCAT^a^		
	CTX‐M‐group 9	CTX‐M‐9 and CTX‐M‐14	CTXMG9‐F	TCAAGCCTGCCGATCTGGT	561	Dallenne et al., 2010
			CTXMG9‐R	TGATTCTCGCCGCTGAAG		
G6	CTX‐M‐group 8/25	CTX‐M‐8, CTX‐M‐25, CTX‐M‐26 and CTX‐M‐39 to CTX‐M‐41	CTXMG8/25‐F	AACRCRCAGACGCTCTAC^a^	326	Dallenne et al., 2010
			CTXMG8/25‐R	TCGAGCCGGAASGTGTYAT^a^		

^a^ Y, T or C; R, A or G; S, G or C; D, A or G or T.

##### Sequence processing

The quality of the raw Sanger sequence was checked by trimming off both ends of sequencing reads using Unipro UGENE v33.0, with a quality threshold of 40. Each consensus sequence was created from the forward and reverse sequence by BioEdit Sequence Alignment Editor version 7.0.5.3 (Hall, 1999). The sequence was aligned using blastn to standard databases/nucleotide collections (nr/nt) and using blastx to non‐redundant protein sequences (nr) at NCBI (cited 10 April 2021). In addition, nucleotide sequences of reference genes were obtained from NCBI (cited 10 April 2021), for manual alignment using blastn at NCBI. The amplicon sequences were also aligned to the genome sequences CP079720‐CP079723 and CP080028.

#### Determination of carbapenemase production

The E‐test MBL MP/MPI (BioMérieux, Sweden AB) was conducted for strain FW 1 and *H. oleronia* DSM 9356^T^. The test strips consisted of a double‐sided 13‐dilution range of only MEM (0.125–8 μg mL^−1^) and MEM (0.032–2 μg mL^−1^) overlaid with a constant concentration of EDTA. MBLs need Zn^2+^ binding in their catalytic centre to hydrolyse β‐lactam antibiotics, and so they are inhibited by chelating agents like ethylenediaminetetraacetic acid (EDTA) (Nordmann & Poirel, 2002). Production of MBLs was observed by differential inhibitory concentrations on two sides of the strip.

#### ARGs and mobile genetic elements annotation

The WGS of strain FW 1 and *H. oleronia* DSM 9356^T^ were annotated using ABRicate (v. 0.8.13). For ARG identification, multiple databases were used: NCBI (24 August 2020), CARD (19 April 2020), ARG‐ANNOT (19 April 2020) and ResFinder (19 April 2020). For plasmid identification, the PlasmidFinder database (24 August 2020) was used. For integron and transposon identification, an integron finder program at Galaxy/Pasteur (https://galaxy.pasteur.fr/) (Néron et al., 2022) and BacAnt (Hua et al., 2021) were used.

### Resistance transferability

Plasmid transformation and conjugation tests were conducted for strain FW 1. For *Bacillus* species, transformation is so far the only way found for HGT (Martinez et al., 1999; Nijland et al., 2010; Spizizen, 1958; Zhang et al., 2014). *Bacillus subtilis* 168 (DSM 402), the most well‐studied strain for transformation recipients in *the* genus *Bacillus* (Martinez et al., 1999; Nijland et al., 2010; Spizizen, 1958; Zhang et al., 2014), was obtained from DSMZ for transformation test. For the conjugation test, *E. coli* K12xB HB101, obtained from the Belgian Co‐ordinated Collections of Micro‐organisms (BCCM), was used as a recipient. This *E. coli* strain is competent and can successfully receive plasmids with a carbapenemase gene, *bla*_IMP‐11_ (Zhao et al., 2012). The *E. coli* strain has resistance to streptomycin (STR), caused by a gene mutation (rpsL20) in a ribosomal subunit (Boyer & Roulland‐Dussoix, 1969), but is sensitive to carbapenems. Since a plasmid associated with TET resistance was identified in strain FW 1, TET‐resistance transferability was investigated together with MEM‐resistance.

#### Plasmid transformation

For strain FW 1, plasmid DNA was extracted by GeneJET Plasmid Miniprep Kit (Thermo Fisher Scientific) according to the manufacturer's instructions. The DNA concentration was quantified by Qubit (Thermo Fisher Scientific), and the fragment length was visualised by agarose gel electrophoresis. The transformation test was conducted according to a previously published protocol (Martinez et al., 1999). Briefly, a full loop (1 μL) of fresh *B. subtilis* 168 was collected from colonies grown overnight on Mueller Hinton agar plates at 37°C. The cells were suspended in 4 mL optimised transformation buffer after which a duplicate of 10 μL plasmid DNA (concentrations 14 and 18.1 ng μL^−1^) was separately added into 1 mL aliquot of the suspended *B. subtilis* 168 culture (Figure S1). After 2‐h incubation on a shaker at 37°C, an aliquot of 100 μL transformation culture was spread evenly on a non‐selection agar plate and selective agar plates containing (a) TET (plate T, 10 μg mL^−1^), (b) MEM (plate M, 2 μg mL^−1^) and (c) both TET and MEM (plate TM, 10 μg mL^−1^, 2 μg mL^−1^). As a growth control, the *B. subtilis* 168 culture without transformation was additionally inoculated on all the agar plates. Under incubation at 37°C, colony growth on all agar plates was checked every 24 h, until up to 72 h. Successful plasmid transformation would show as colony growth on selective agar plates for the transformed culture while not for the culture of *B. subtilis*.

#### Plasmid conjugation

##### Plate mating conjugation

The full workflow for the conjugation tests is shown in Figure S2. In brief, strain FW 1 was streaked on selection plates containing only TET (plate T, 5 μg mL^−1^), only MEM (plate M, 10 μg mL^−1^), both TET and STR (plate TS, 5 μg mL^−1^, 30 μg mL^−1^) and both MEM and STR (plate MS, 10 μg mL^−1^, 30 μg mL^−1^). *E. coli* HB101 was streaked on selection plates containing only STR (plate S, 30 μg mL^−1^) and on TS and MS plates. These plates were incubated at 37°C in darkness for 1–4 days, depending on observed colony growth. Strain FW 1 from plates T and M was cross‐streaked with *E. coli* HB101 separately on two non‐selection plates (plate N). After incubation, bacterial cell equivalents of 2–3 colonies were picked at five crossing points from each plate N and streaked, respectively, onto selection plate TS or MS. The selection plates were incubated for up to 4 days at 37°C for growth observation. If any growth was seen on the selection plates, representative colonies were picked and re‐streaked on both TS and MS plates, and incubated for 1 day at 37°C. Successful plasmid conjugation was assumed if colony growth was found on TS and MS plates. However, to confirm the plasmid conjugation, colonies growing on these plates were subjected to 16S rRNA sequencing, following the protocol in Sun, Bjerketorp, et al. (2020), to confirm their taxonomic identity. Antimicrobial susceptibility testing (AST) was also performed on the transconjugants, to determine their resistance to MEM, TET and STR.

##### Broth mating conjugation

Strain FW 1 and *E. coli* HB101 were incubated separately in 5 mL MHB in 15 mL sterile Falcon tubes inclined with shaking at 150 rpm, for 1 day at 37°C in darkness (Figure S2). Two cultures were combined in a total volume of 5 mL in a ratio of 1:1 in terms of bacterial amount determined by optical density. After brief vortexing, the mixed culture was incubated for 1 day at 37°C. An aliquot of 75 μL of the mixed culture was spread and quadrantally streaked on the selection plates TS and MS. The selection plates were incubated for 1 day at 37°C to check for growth. Taxonomic identification and AST for the colonies were performed according to the same procedure as used for the plate mating conjugation (Section ‘Plate mating conjugation’).

## RESULTS

### Strain FW 1 identification and characterization

#### Comparison of genotypic characteristics

**FIGURE 1:**
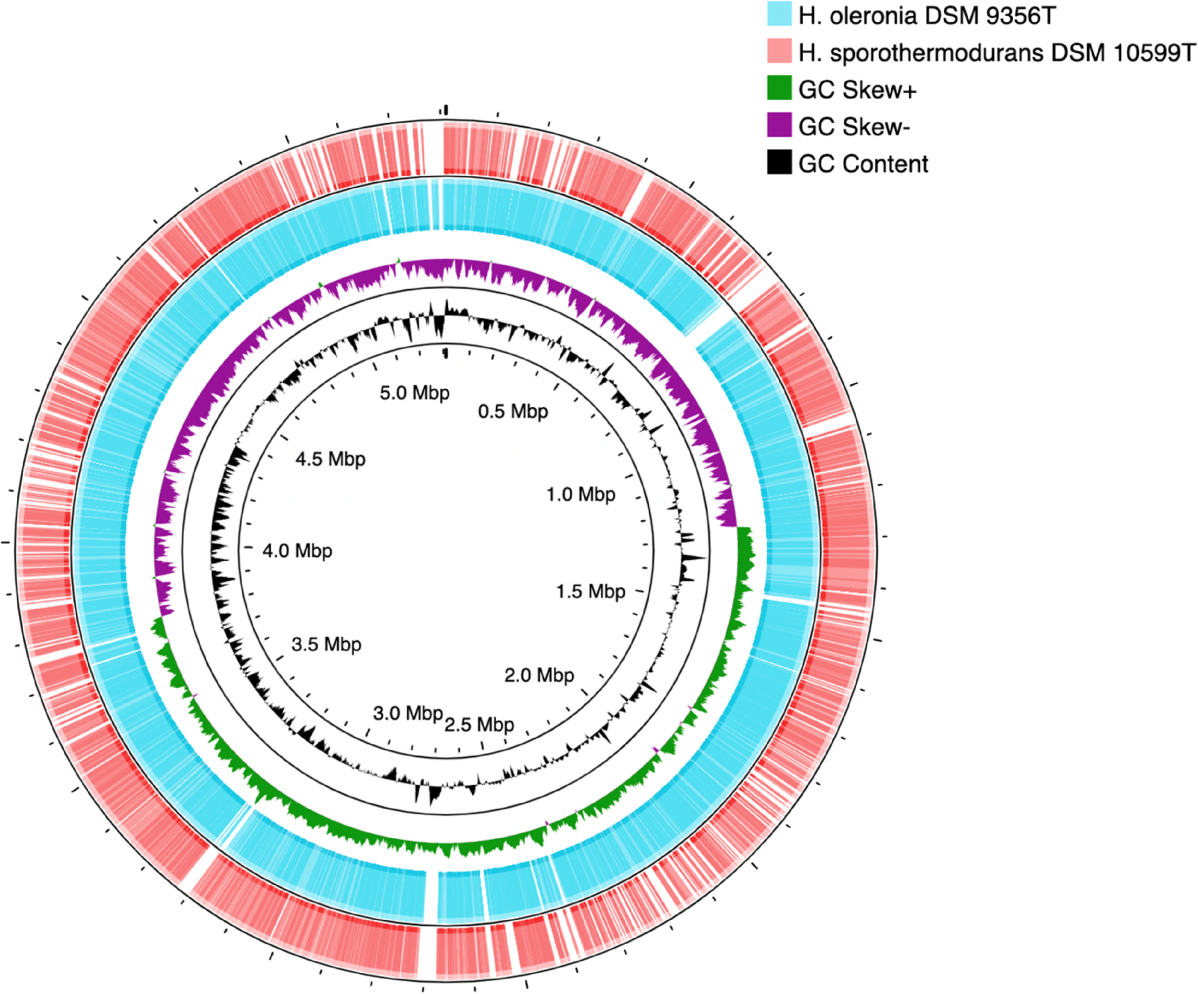
Comparison of the genome of strain FW 1 to that of *H. oleronia* DSM 9356^T^ and *H. sporothermodurans* DSM 10599^T^. The innermost ring is FW 1 with a genome size ruler, followed by its GC content and GC skew rings. The outermost two rings are DSM 9356^T^ and DSM 10599^T^with genome alignment to FW 1, where coloured zones indicate matches and blank zones indicate mismatches.

According to 16S rRNA gene sequence comparisons, strain FW 1 (1460 bp, accession no. NR_043325.1) shared 97.88% and 96.44% similarity with *H. oleronia* DSM 9356^T^ and *H. sporothermodurans* DSM 10599^T^, respectively. For WGS, the GC content of strain FW 1 and *H. oleronia* DSM 9356^T^ was 35.1% in both cases, while that of *H. sporothermodurans* DSM 10599^T^ was 35.6%. WGS similarity was measured by OrthoANI and in silico DDH values, and visualised by Proksee (Figure 1). The OrthoANI value for strain FW 1 with *H. oleronia* DSM 9356^T^ and *H. sporothermodurans* DSM 10599^T^ was 98.23% and 76.70%, respectively. For DDH values, according to the recommended formula (identities/HSP length), strain FW 1 shared a similarity of 84.10% and 22.60% with *H. oleronia* DSM 9356^T^ and *H. sporothermodurans* DSM 10599^T^, respectively. Genome comparison, as visualised in Figure 1, showed that *H. oleronia* DSM 9356^T^ had good and almost complete genome alignment to strain FW 1, while several mismatches were observed with *H. sporothermodurans* DSM 10599^T^.

#### Comparison of phenotypic characteristics

Strain FW 1 and *H. oleronia* DSM 9456^T^ were further compared by phenotypic characterisation using API 50CH and API 20E tests (Table 2), and other tests such as gram‐staining, cell growth conditions and morphology (Table 3). The test results showed them to be highly similar, still with slight differences for optimal growth conditions, that is, temperature and pH.

**TABLE 2:** Characterisation of strains *Bacillus* sp. FW 1 and *Bacillus oleronius* DSM 9356^T^, using the API 50CH and API 20E tests.

Kit	Test	FW 1	DSM 9356^T^
API 50CH	Glycerol, D‐ribose, D‐glucose, D‐fructose, D‐mannitol, *N*‐acetylglucosamine, esculin ferric citrate, D‐cellobiose, D‐maltose, D‐trehalose, D‐tagatose	+	+
	Erythritol, D‐arabinose, L‐arabinose, D‐xylose, L‐xylose, adonitol, methyl‐beta‐D‐xylopyranoside, D‐galactose, D‐mannose, L‐sorbose, L‐rhamnose, dulcitol, Inositol, D‐sorbitol, methyl‐alpha‐D‐mannopyranoside, methyl‐alpha‐D‐glucopyranoside, amygdalin, arbutin, salicin, D‐lactose (bovine origin), D‐melibiose, sucrose, inulin, D‐melezitose, D‐raffinose, starch, glycogen, xylitol, gentiobiose, D‐turanose, D‐lyxose, D‐fucose, L‐fucose, D‐arabitol, L‐arabitol, potassium gluconate, potassium 2‐ketogluconate, potassium 5‐ketogluconate, ß‐galactosidase	−	−
API 20E	Voges–Proskauer, Gelatinase	+	+
	Arginine dihydrolase, lysine decarboxylase, ornithine decarboxylase, citrate utilisation, H2S production, urease, tryptophan deaminase, indole production	−	−

*Note*: Tests performed with API 50CH and some additional tests with API 20E according to the manufacturer's instructions. +, positive; −, negative.

**TABLE 3:** Phenotypic characteristics of strain FW 1 and *H. oleronia* DSM 9356^T^.

Characteristic	FW 1	DSM 9356^T^
Gram staining	–	–
Cell growth		
Temperature range (optimum), °C	30–37(30)	20–37(37)
pH range (optimum)	6–11(9)	6–11(8)
Anaerobic growth	–	–
Spreading growth	–	–
Cell morphology		
Form	Circular	Circular
Size (mm)	0.2–1.5	0.6–2
Elevation	Raised	Raised
Margin	Lobate	Lobate
Surface	Rough	Rough
Opacity	Opaque	Opaque
Colour	Buff	Buff
Antibiotic susceptibility		
Ampicillin	R	R
Ceftazidime	R	R
Meropenem	R	I
Vancomycin	S	S
Ciprofloxacin	S	S
Rifampicin	S	S
Chloramphenicol	S	S
Clindamycin	S	S
Erythromycin	S	S
Tetracycline	R	S
Gentamicin	S	S
Sulfamethoxazole/trimethoprim	S	S

*Note*: R, I and S represent resistant, intermediate resistant and susceptible, respectively. The minimum inhibitory concentrations of strain FW1 and DSM 9356T for the antibiotics can be found in Table S1.

### Mechanism of carbapenem resistance

#### PCR mapping of carbapenem resistance genes

Mapping of carbapenemase and ESBL genes for strain FW 1 and *H. oleronia* DSM 9356^T^ was carried out using multiplex and single‐plex PCR assays (Table 1). In gel electrophoresis, amplicons were observed for group G3, but only for strain FW 1 and for not *H. oleronia* DSM 9356^T^. This result was followed up by a further single‐plex PCR array with primers VIM, IMP and KPC separately, to evaluate the presence of specific resistance genes within this group. Amplicons were only obtained for VIM primers. The amplicon size obtained was precisely as designed, 390 bp in length after quality control. However, the nucleotide sequence (Sanger) of the retrieved product showed no significant similarity with sequences in NCBI using BLAST, even at a least similar level. In addition, no significant similarity was found between the amplicon and VIM‐1, VIM‐2 and VIM‐7 after manual alignment. Translation of the amplicon sequence into hypothetical proteins showed some similarities with proteins from species belonging to *Bacillus*, for example, *B*. sp. Gen3 (coverage 97%, similarity 90.62%, accession no. WP_180213886.1) and *Bacillus farraginis* (coverage 97%, similarity 89.84%, accession no. WP_058002202.1).

#### MBL production

Production of MBL was observed for strain FW 1 and *H. oleronia* DSM 9356^T^. For both strains, the MIC value decreased from 8 to 0.032 μg mL^−1^ when MEM was used together with EDTA.

#### ARGs and mobile genetic elements annotation

**FIGURE 2:**
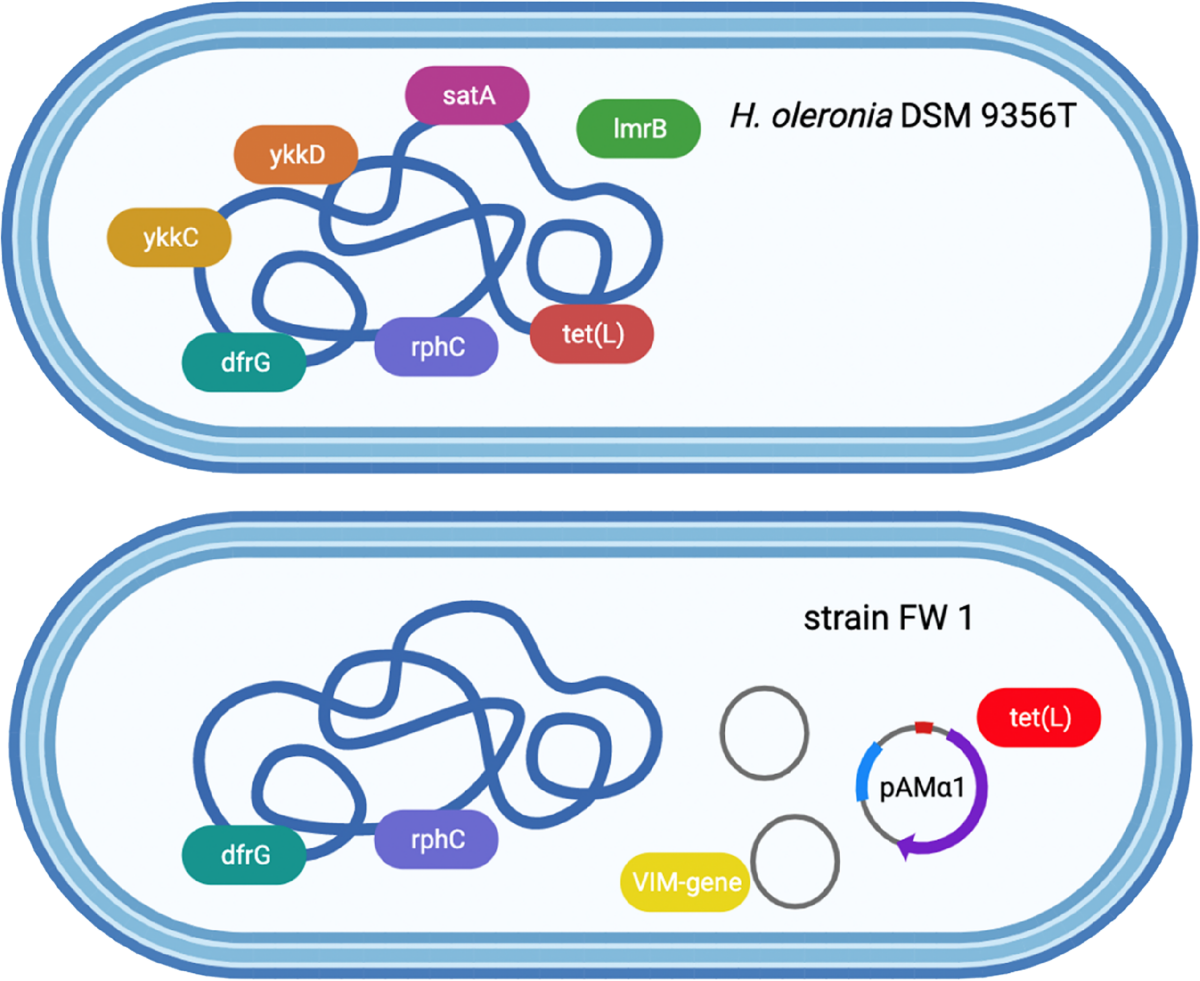
Antibiotic resistance genes (ARGs) and plasmid carried by strain FW 1 (accession no. CP079720 for chromosome, CP079721–CP079723 for ecDNA) and *H. oleronia* DSM 9356^T^ (accession no. CP080028). ARGs were identified in the genome sequences using the NCBI, CARD, ARG‐ANNOT and ResFidner databases. The plasmid in strain FW 1 was identified using the PlasmidFinder database. Two unknown circular ecDNAs were observed for strain FW 1 using the tool Bandage (version 0.8.3).

No carbapenem resistance genes were found in the genome sequences of strain FW 1 and *H. oleronia* DSM 9356^T^, but some other ARGs were identified (Figure 2). For the type strain, *H. oleronia* DSM 9356^T^, seven ARGs were found in its chromosome, encoding for resistance to trimethoprim (*dfr*G), rifampicin (*rph*C), TET (*tet*L, *ykk*C and *ykk*D), lincosamide (*lmr*B), streptothricin (*sat*A) and aminoglycoside and phenicol (*ykk*C and *ykk*D). In comparison, only two genes (*dfr*G and *rph*C) of the seven were found in the chromosome of strain FW 1. However, three circular ecDNAs were found in FW 1, but not present in the type strain. One ecDNA was identified as plasmid pAMα1, carrying three copies of gene *tet*L. The other two ecDNAs were not classified. However, it is worth noting that the sequence of amplicon obtained from PCR amplification via the VIM primers was found in one of these two ecDNAs. In addition, no integrons and transposons were found in either genome of FW 1 or DSM 9356^T^.

### MEM resistance transferability

#### Plasmid transformation

After 3 days incubation, no growth was found on any selection agar plates, neither for the original *B. subtilis* 168 culture nor for the culture incubated with plasmid DNA isolated from strain FW 1. However, growth was observed on the non‐selection plates for both cultures.

#### Plasmid conjugation

After agar mating conjugation of strain FW 1 and *E. coli* HB 101, colony growth was observed on selection plate TS, but no growth was observed on selection plate MS (Figure S2). As expected, no growth was found for non‐mated strain FW 1 and *E. coli* HB 101 on selection plates TS and MS. The colonies from selection plate TS were subculturable on both TS and MS. Similar results were found in broth mating conjugation, in that colony growth was observed on plate TS.

Sub‐cultured colonies from selection plates TS and MS in agar mating conjugation were randomly isolated and identified by amplification of 16S rRNA gene followed by Sanger‐sequencing. However, both *H. oleronia* and *E. coli* were subsequently identified. A further AST was conducted for these two strains. They displayed the same resistance characteristics as before conjugation, that is, FW 1 was resistant to MEM and TET, but sensitive to STR, while *E. coli* HB 101 was susceptible to MEM and TET, but resistant to STR (Table 4). In conclusion, no successful conjugants with elevated resistance were obtained from agar mating or broth mating conjugation.

**TABLE 4:** Minimum inhibitory concentrations (μg mL^−1^) of meropenem (MEM), tetracycline (TET) and streptomycin (STR) for *H. oleronia* FW 1 and *E. coli* HB 101 before and after conjugation, determined using the E‐test method.

Sample	MEM		TET		STR	
	Before	After	Before	After	Before	After
FW 1	32/32/32	32/32/32	96/64/96	96/96/96	8/12/8	8/8/12
HB 101	0.02/0.02/0.02	0.02/0.02/0.02	1.50/1.50/2	2/2/2	1024/1024/1024	1024/1024/1024

## DISCUSSION

### Strain FW 1 identification

In the WGS comparison, a noticeable difference in similarity to strain FW 1 was observed between *H. oleronia* and *H. sporothermodurans*. For ANI values, approximately 95%–96% is considered the species boundary (Chun & Rainey, 2014). For DDH values, two respective organisms are regarded as distinct species if DDH similarity is below 70% (Stackebrandt & Goebel, 1994). The similarity between strain FW 1 and *H. oleronia* in the different methods applied in this study was higher than the corresponding threshold in all cases, while the similarity obtained for strain FW 1 compared with *H. sporothermodurans* was not even close to the threshold. Therefore, strain FW 1 is suggested to be taxonomically assigned to *H. oleronia*, based on WGS similarity. This taxonomic classification was confirmed by a comparison of phenotypic characteristics, including API tests, cell morphology and so forth, in which strain FW 1 and *H. oleronia* exhibited highly similar characteristics, with some small differences. It is worth noting that strain FW 1 and *H. oleronia* DSM 9356^T^ could not grow at 55°C, which indicates that thermophilic digestion can be an effective way to mitigate MEM resistance associated with strain FW 1. However, clear differences were seen between strain FW 1 and *H. oleronia* DSM 9356^T^ for antibiotic resistance to MEM and TET, which in both cases were higher for strain FW 1. These differences between the strains could have been caused by gene mutations or foreign gene acquisition. In general, antibiotic resistance pattern is not a determining factor for the taxonomic classification of bacteria (Tindall et al., 2010). In this study, both genotypic and phenotypic characteristics indicated that strain FW 1 should be taxonomically positioned to *H. oleronia*.

### Mechanism of carbapenem resistance

#### Detection of carbapenemase genes by PCR

In terms of carbapenem hydrolysis and geographical spread, the most effective carbapenemases are KPC, VIM, IMP, NDM and OXA‐48 types (Meletis, 2016). In this study, except for NDM, all these enzymes and other ESBL were covered in the analyses. Among the 15 gene groups targeted, amplicons were obtained only by the primers targeting VIM family genes, and only for strain FW 1. The VIM primers used in this study, together with other four pairs of primers targeting the IMP, SPM, GIM and SIM families, were initially designed in multiplex PCR for rapid detection of genes encoding acquired MBLs (Ellington et al., 2007). The primers have been widely used, particularly for the detection of MBLs in pathogenic organisms, such as *Pseudomonas* spp., *Klebsiella* spp. and *Acinetobacter* spp. (Ellington et al., 2007). The VIM primers have also been used for strains of *B. cereus*, with 21.2% of 66 strains analysed found to carry *bla*_VIM_‐like genes with 99% similarity to *bla*_VIM‐1_, *bla*_VIM‐35_ and *bla*_VIM‐33_, typically identified in *Pseudomonas*, *Klebsiella* spp. and *E. coli* (Torkar & Bedenić, 2018)). In this study, however, the amplicon sequence amplified by the VIM primers was not significantly similar to any *blaVIM‐like* genes, even though the amplicon size was precisely as expected. Besides, proteins from other *Bacillus* strains, similar to the hypothetical protein from translation of the amplicon sequence, appeared not to have any motifs related to MBL or other β‐lactamase production, according to their conserved domains found at NCBI. Therefore, the amplified sequence could have been produced by specificity deficiency of the VIM primers, and the sequence region with no carbapenemase gene happened to be captured. In brief, no carbapenemase genes were identified by PCR mapping with the primers investigated in this study.

#### MBL production

MBLs have a broad substrate spectrum and can catalyse hydrolysis of virtually all β‐lactam antibiotics even carbapenems, except for monobactams (Palzkill, 2013). In this study, both strain FW 1 and *H. oleronia* DSM 9356^T^ were found to produce MBL, which could explain their resistance to AMP and CAZ, and the intermediate resistance of *H. oleronia* DSM 9356^T^ to MEM (8 μg mL^−1^). The higher MEM resistance of strain FW 1 (32 μg mL^−1^) might be caused by other MBL or carbapenemase genes not present in the type strain. Among gene groups encoding for MBL production, in this study, VIM‐ and IMP‐type genes were investigated as representatives, because of their frequent occurrence and effectiveness in carbapenem hydrolysis (Meletis, 2016). Considering that the only VIM‐amplicon obtained in this study was unlikely to encode for MBL production, the possibility of conferring MEM resistance to strain FW 1 by VIM‐ and IMP‐type genes could be excluded. Therefore, the high MEM resistance was possibly caused by less common MBL genes other than VIM‐ and IMP‐likes, for example, SPM, GOB and SIM, typically identified in *Pseudomonas aeruginosa*, *Chryseobacterium meningoseptica* and *Acinetobacter baumannii*, respectively (Palzkill, 2013). These possibilities are further discussed below in relation to the results of genome analysis.

#### Genome analysis

Inconsistency was found between genotypic and phenotypic characteristics in terms of antibiotic resistance. First, no gene encoding known or described β‐lactamases (BLs) was found for strain FW 1 and *H. oleronia* DSM 9356^T^, which is in line with the PCR mapping results that no carbapenemase and ESBL genes were detected. However, it was not consistent with their phenotypic resistance to AMP, CAZ and MEM, which was confirmed by E‐test (Table S1). Additionally, MBL production was found for both strains, but corresponding genes were not identified in either strain. Therefore, the genes encoding for MBL production could be novel, supporting suggestions in a previous study (Brandt et al., 2017). Analysis of BLs based on protein sequence databases has revealed that a substantial number of unknown and functionally uncharacterised BLs exist in a multitude of environmental and pathogenic species (Brandt et al., 2017). Second, the ARGs identified in this study were not expressed in corresponding phenotypic resistance. For example, both strains carried a gene, *rph*C, but neither exhibited rifampicin resistance according to the E‐test. (Table S1). This discrepancy might be related to the silencing of gene expression. A previous study found that, although resistance genes and their promoters were intact, they were not expressed (Enne et al., 2006). Similarly, a study of *B. anthracis* found that, because of poor gene transcription, gene expression did not reach sufficient levels to confer resistance to β‐lactam agents (Chen et al., 2003).

#### Speculation of the mechanism of resistance

Resistance to carbapenems in some species is intrinsic. For example, *Stenotrophomonas maltophilia* possesses the endogenous MBL L1 (Sánchez & Martínez, 2015). However, intrinsic resistance to carbapenems is not common among clinically important bacteria and for most of them the resistance is acquired by gene mutations or HGT (Meletis, 2016). The type strain in this study, *H. oleronia* DSM 9356^T^, was originally isolated from the hindgut microbiota of the termite *Reticulitermes santonensis* (Kuhnigk et al., 1995), which was unlikely exposed to carbapenem pressure. However, this strain still exhibited intermediate resistance to MEM. In contrast, strain FW 1 isolated from an environment likely to have a higher carbapenem pressure showed comparably higher resistance. It could therefore be hypothesised that the intermediate resistance in the type strain is intrinsic, while the elevated resistance for strain FW 1 is caused by gene mutations or acquisition of foreign genes. Generally, Gram‐negative bacteria become carbapenem‐resistant by recruiting one or more out of three mechanisms, (a) expulsion of carbapenems after their entrance, (b) diminishing the permeability of outer membrane and (c) production of carbapenemases (Meletis, 2016). The expulsion of carbapenems is mediated by a tripartite efflux pump system composed of a protein transporter in the cytoplasmic membrane, a periplasmic connective protein and an outer membrane porin (Schweizer, 2003). In this study, since no genes corresponding to such proteins were found in the genomes of either strain FW 1 or DSM 9356^T^, the resistance was not likely caused by the mechanism (a). As for (b), the alterations of porins can contribute to blocking the penetration of carbapenems into the bacterial cell (Bialek‐Davenet et al., 2017). This mechanism could potentially explain the elevated resistance for FW 1 due to porins change. Such porin mutations typically do not have the potential for mobilisation to other bacteria (Walsh, 2000). Carbapenemase production is the main resistance mechanism among the three identified carbapenem‐resistant bacteria (Meletis, 2016). It is also the most likely mechanism of MEM resistance for strain FW 1 and DSM 9356^T^, as both of them were observed to produce MBLs. Considering that no carbapenemase genes were found by genome analysis and PCR mapping, the higher resistance to MEM for strain FW 1 was therefore most probably caused by the production of unclassified carbapenemase, or overexpression of MBL as compared to strain DSM 9356^T^. Moreover, the elevated resistance to MEM for strain FW 1 was most likely caused by gene mutations, since no integrons and transposons were found in the genome of FW 1, and the plasmid pAMγ1 was analysed to be not likely related to MEM resistance.

### Resistance transferability

#### Genome analysis

Based on the results of our WGS analysis, the transferability of MEM resistance can be preliminarily excluded, since no integron or transposon was identified. The only identified plasmid pAMα1 carrying *tet*L was originally found in *Streptococcus faecalis* strain DS 5 (Clewell et al., 1974). Plasmid pAMα1 is not conjugative, but it is mobilisable by co‐resident conjugative elements, that is, plasmid pAMγ1 (Dunny & Clewell, 1975). Although pAMγ1 was not found for strain FW 1 based on WGS analysis, the two unclassified ecDNAs found in this strain could potentially assist its transfer. If the unknown carbapenemase genes happen to be located in these ecDNAs and are transferable, there could be a possibility of resistance transfer.

#### Plasmid transformation/conjugation

Transformation/conjugation tests indicated that neither the MEM resistance nor the TET resistance for strain FW 1 was transferable to *B. subtilis* 168 or *E. coli* HB 101. However, it is worth noting that in the conjugation tests, strain FW 1 and *E. coli* HB 101 were able to grow on selection plate TS without acquiring new resistance. This might be due to unknown interactions between the two strains allowing them to benefit each other and survive the antibiotic pressure of TET and STR. Therefore, taking both the genome analysis and plasmid transformation/conjugation results into consideration, MEM resistance is not likely to be transferable for strain FW 1. In addition, since TET resistance was not transferred via plasmid transformation/conjugation, the two unclassified ecDNAs are presumably unable to facilitate pAMα1 transfer.

## CONCLUSIONS

The MEM‐resistant strain *Bacillus* sp. FW 1 found in biogas digestate should be assigned to *H. oleronia* (previous *Bacillus oleronius*), based on similarities in both WGS and phenotypic characteristics. MBL production was confirmed for both strain FW 1 and *H. oleronia* DSM 9356^T^, which to some extent explained the intermediate resistance to MEM of strain DSM 9356^T^. The higher MEM resistance seen for strain FW 1 is most probably linked to the production of unidentified carbapenemase or overexpression of MBL as compared to strain DSM 9356^T^. The masked mechanism of resistance indicates the importance of further characterisation studies on novel genes associated with the bacterial community in digestate and other environments. Most importantly, MEM resistance was suggested to be non‐transferable for strain FW 1, based on both genome analysis and plasmid transformation/conjugation tests. Therefore, the MEM‐resistant strain *H. oleronia* FW 1 can be assumed to represent a limited risk of MEM resistance spread within biogas digesters and to the environment when digestate is applied to arable land. Moreover, thermophilic digestion may further mitigate the risk, as *H. oleronia* FW 1 could not grow at 55°C.

## AUTHOR CONTRIBUTIONS

**He Sun:** Formal analysis (equal); investigation (equal); writing – original draft (equal). **Jolanta J. Levenfors:** Supervision (equal). **Christian Brandt:** Formal analysis (equal). **Anna Schnürer:** Funding acquisition (equal); project administration (equal); supervision (equal); writing – review and editing (equal).

## CONFLICT OF INTEREST STATEMENT

The authors declare no conflicts of interest.

## Supporting information


**DATA S1:** Supporting Information.

## Data Availability

Data available on request from the authors.
