# Supplementary material for: Characterisation of meropenem‐resistant Bacillus sp. FW 1 isolated from biogas digestate
Source: Environ Microbiol Rep. 2023 Nov 15;16(1):e13217. doi: 10.1111/1758-2229.13217 (PMC10866066; doi:10.1111/1758-2229.13217)
Supplement: Supplementary file 1 — DATA S1: Supporting Information. [file EMI4-16-e13217-s001.docx]

# Supplementary Materials

Table S1. Minimum inhibitory concentrations (μg mL^-1^) of strain FW 1 and *H. oleronia* DSM 9356, using the E-test method for: ampicillin (AMP), ceftazidime (CAZ), meropenem (MEM), vancomycin (VAN), ciprofloxacin (CIP), rifampicin (RIF), chloramphenicol (CHL), clindamycin (CLI), erythromycin (ERY), tetracycline (TET), gentamicin (GEN) and sulfamethoxazole/trimethoprim (SXT).

| Strain | AMP | CAZ | MEM | VAN | CIP | RIF | CHL | CLI | ERY | TET | GEN | SXT |
| --- | --- | --- | --- | --- | --- | --- | --- | --- | --- | --- | --- | --- |
| *B.* sp. FW 1 | 3 | 128 | 32 | 2 | 0.13 | 0 | 2 | 0.5 | 1 | 96 | 0.02 | 0.05 |
| *B. oleronius* DSM 9356 | 12 | 256 | 8 | 1.5 | 0.25 | 0.32 | 4 | 0.19 | 0.38 | 4 | 0.05 | 0.05 |


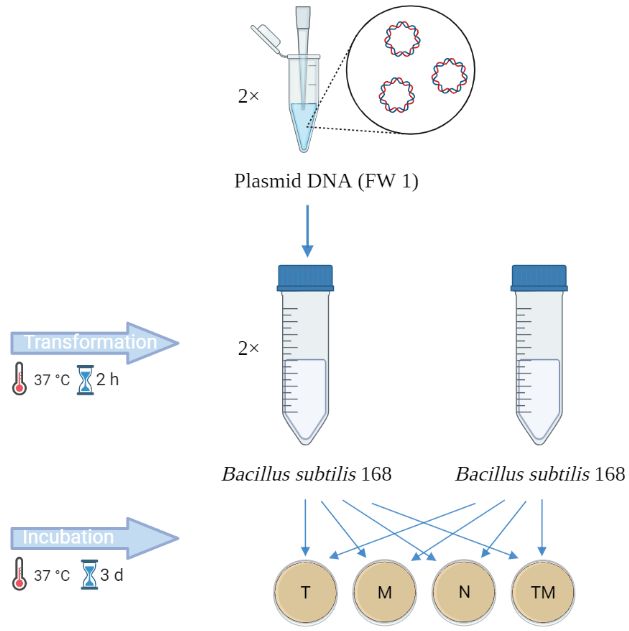


Fig. S1. Schematic workflow of plasmid transformation. N represents non-selection plates. T, M, and TM represent selection plates containing TET, MEM, and TET+MEM, respectively.


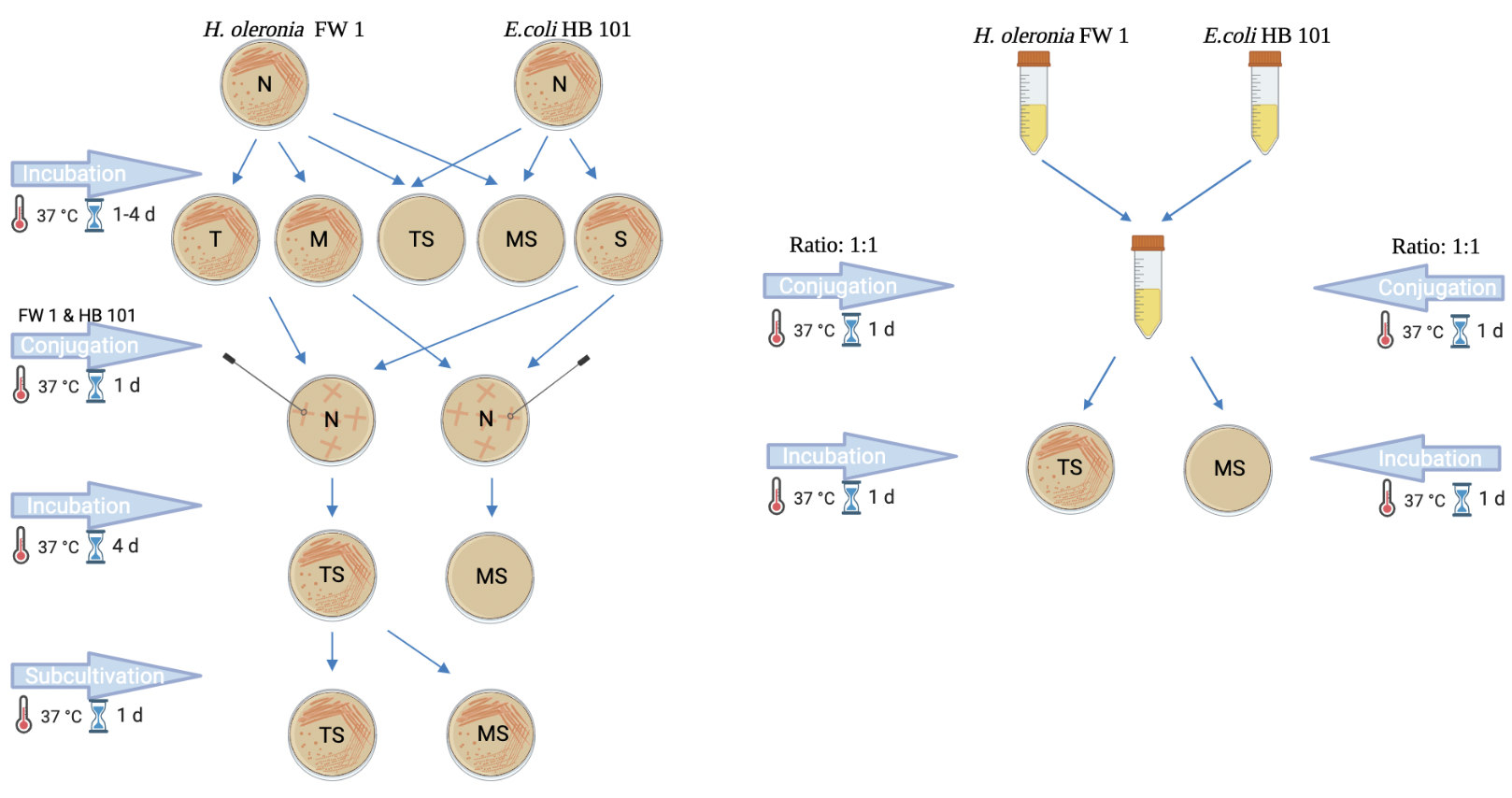


Fig. S2. Schematic workflow of agar and broth mating conjugation. N represents non-selection plates. T, M, and S represent selection plates containing TET, MEM, and STR, respectively, and TS and MS represent selection plates containing TET + STR and MEM + STR, respectively.
